# Inflammation, DNA Damage, *Helicobacter pylori* and Gastric Tumorigenesis

**DOI:** 10.3389/fgene.2017.00020

**Published:** 2017-02-27

**Authors:** Polyxeni Kalisperati, Evangelia Spanou, Ioannis S. Pateras, Penelope Korkolopoulou, Anastasia Varvarigou, Ioannis Karavokyros, Vassilis G. Gorgoulis, Panayiotis G. Vlachoyiannopoulos, Stavros Sougioultzis

**Affiliations:** ^1^Gastroenterology Unit, Department of Pathophysiology, School of Medicine, National and Kapodistrian UniversityAthens, Greece; ^2^Department of Histology and Embryology, School of Medicine, National and Kapodistrian UniversityAthens, Greece; ^3^1st Department of Pathology, Laiko Hospital, School of Medicine, National and Kapodistrian University of AthensAthens, Greece; ^4^Department of Pediatrics, University of Patras Medical SchoolPatras, Greece; ^5^1st Department of Surgery, Laiko Hospital, University of Athens, School of MedicineAthens, Greece; ^6^Biomedical Research Foundation of the Academy of AthensAthens, Greece; ^7^Faculty of Biology, Medicine and Health Manchester Cancer Research Centre, Manchester Academic Health Sciences Centre, The University of ManchesterManchester, UK; ^8^Department of Pathophysiology, School of Medicine, National and Kapodistrian UniversityAthens, Greece

**Keywords:** *Helicobacter pylori (H. pylori)*, double strand breaks (DSBs), DNA damage repair (DDR), γH2AX, eradication, genomic instability, tumorigenesis, chronic inflammation

## Abstract

*Helicobacter pylori (H. pylori)* is a Gram negative bacterium that colonizes the stomach of almost half human population. It has evolved to escape immune surveillance, establishes lifelong inflammation, predisposing to genomic instability and DNA damage, notably double strand breaks. The epithelial host cell responds by activation of DNA damage repair (DDR) machinery that seems to be compromised by the infection. It is therefore now accepted that genetic damage is a major mechanism operating in cases of *H. pylori* induced carcinogenesis. Here, we review the data on the molecular pathways involved in DNA damage and DDR activation during *H. pylori* infection.

## Introduction

Rudolf Virchow was the first to propose a potential association between chronic inflammation and cancer, based on his observations of inflammatory cells presence in human malignant tissues. Eventually, inflammation was established as an important hallmark of cancer (Hanahan and Weinberg, 2011). Approximately, 20–25% of cancer cases are attributed to chronic inflammation (Ames et al., 1995; Mantovani et al., 2008).

Accumulating evidence suggest that chronic inflammation either non-infectious such as in autoimmune disorders, or as a result of pathogen infection is connected to cancer development. Characteristic examples of autoimmune disorders that may promote tumorigenesis are celiac disease and Sjögren syndrome (Austad et al., 1967; Voulgarelis et al., 2012). Several infectious agents have been associated with carcinogenesis including hepatitis B and C viruses, strongly associated with hepatocellular carcinoma, human papilloma virus (HPV) with cervical cancer and *H. pylori* with gastric cancer (Plummer et al., 2016). Infection-related cancer is caused either by direct tumorigenic effect of the pathogen, or indirectly through the accompanying inflammation and the subsequent genomic instability (Cortes-Bratti et al., 2001; Coussens and Werb, 2002; Scanu et al., 2015).

Inflammation as well as several endogenous or exogenous agents, may result in DNA lesions which in turn promote DNA repair pathways and interception of DNA replication, a mechanism known as replication stress (Berti and Vindigni, 2016). In particular, inflammation induces a vicious cycle among continuous injury and repair, resulting in aberrant cell proliferation that favors replication stress. Inflammation-induced replication stress enhances DNA damage, which subsequently induces DNA damage response (DDR), genomic instability and finally tumorigenesis (Coussens and Werb, 2002).

Herein, we review the interplay between pathogens and especially *H. pylori*, inflammation, genomic instability, and tumorigenesis.

## Double Strand Breaks (DSBs) and DNA Damage Response (DDR) Pathway-Overview

Human cells and their genome are under constant attack by DNA-damaging agents, resulting in 10s of 1000s of DNA lesions daily (Lindahl and Barnes, 2000). DSBs are lethal and difficult to repair (Khanna and Jackson, 2001; Rouse and Jackson, 2002; Harper and Elledge, 2007; Jackson and Bartek, 2009).

The cell responds to DNA lesions by activating a complex mechanism, named DDR, which detects and then initiates signaling in order to repair DNA (Harrison and Haber, 2006; Harper and Elledge, 2007). There are several DDR pathways, according to the type of DNA lesion. As far as DSBs are concerned, non-homologous end-joining (NHEJ) and homologous recombination (HR), are the major DNA repair mechanisms (Lieber, 2008; San Filippo et al., 2008).

Double strand breaks are detected by the DNA damage sensors namely MRN complex (Mre11-Rad50-Nbs1) which in turn orderly recruits other agents of the DDR pathway (Lee and Paull, 2005; Jackson and Bartek, 2009; Polo and Jackson, 2011; Roos and Kaina, 2013).

In brief, DSBs promote activation of ATM and ATR, resulting in H2AX histone phosphorylation at Ser 139 (γH2AX) an initial step toward DNA repair (Shiloh, 2003; Bartek and Lukas, 2007; Cimprich and Cortez, 2008; Cook et al., 2009; Xiao et al., 2009). γH2AXformation activates the transducers Chk1 and Chk2 leading to p53 activation (Kastan and Bartek, 2004; Bartek and Lukas, 2007; Riley et al., 2008). As soon as p53 is activated, cell cycle arrest is triggered, in order for the DNA lesion to be repaired. Alternatively, if the lesion cannot be repaired apoptosis or premature senescence are promoted. All the above p53-induced responses are tumor suppressing.

A novel model of tumorigenesis was proposed by Bartkova et al. (2005) and Gorgoulis et al. (2005), suggesting that DNA replication stress enhance DSBs formation, leading to genomic instability and selective pressure for p53 mutations, abrogating the tumor suppressing actions of p53 (Halazonetis et al., 2008).

If the DDR pathway is ineffective either by overload or p53 mutations, faithful DNA repair is compromised resulting in genomic instability (Negrini et al., 2010). Notably, the DDR pathway, through its upstream kinase ATM, also keeps in check another major tumor suppressor factor, namely p14^ARF^ (Velimezi et al., 2013; Wallace et al., 2014) that functions as a second anti-tumor barrier to DDR activation (Evangelou et al., 2013). Thus p53 inactivation can have detrimental effect(s) since both these anti-tumor routes are compromised.

## Inflammation, Genomic Instability, and Tumorigenesis

Chronic inflammation results in inflammatory cell infiltration and production of cytokines in tissues. Inflammatory cells of innate immunity such as macrophages, MDSCs, neutrophils, and dendritic cells are known to present tumor enhancing activity (Gabrilovich and Nagaraj, 2009; Grivennikov et al., 2010; Terzic et al., 2010).

Recently, Pereira-Lopes et al. (2015) reported that macrophage functions, such as proliferation, ROS and cytokine production, are mediated through DDR and in particular, by the NBS1 protein, part of the MRN sensor complex. They studied mice carrying NBS1 hypomorphic protein and found impairment of DDR, accumulation of DSBs, defects in macrophage proliferation, increased production of cytokines and increased rate of macrophage senescence. Taken together their findings suggest the role of DDR in controlling immunopathology and facilitating tissue repair during inflammation and infections (Colonna, 2015).

Inflammatory tissues contain several growth factors, such as epidermal growth factor (EGF), platelet derived growth factor (PDGF), fibroblast growth factor (FGF), TGF-α, TGF-β, insulin-like growth factors 1 and 2 that directly promote cell proliferation, replication stress and eventually DSBs formation (Cianfarani et al., 1998; Jakowlew, 2006; Aivaliotis et al., 2012). In addition, cytokines such as IL-1β, IL-6, TNF-α and IFN-γ induce the formation of RONS that trigger DNA mutations and epigenetic alterations affecting the proteins responsible for cell cycle control or survival (Colotta et al., 2009; Jurk et al., 2014; Kiraly et al., 2015).

A characteristic example of the interaction between inflammation, cytokines and DDR is the SASP. SASP refers to the production of various substances by the senescent cell, including inflammatory cytokines (e.g., IL-6, IL-8), proteases and growth factors (Bavik et al., 2006; Coppe et al., 2008; Malaquin et al., 2013) that act as inflammatory stimulators and affect neighboring non-senescent cells, in a paracrine fashion. Depending on the microenvironment, SASP may act either as anti-tumorigenic factor favoring senescence, or as oncogenic stimulant (Gorgoulis and Halazonetis, 2010). Specifically, SASP factors enhance senescence in normal and low grade preneoplastic cells but promote tumor development in high grade preneoplastic or cancerous cells. SASP is considered a delayed response, taking days to operate and is driven by DDR which is constantly active in senescent cells. It is a means of long-term modulation of the microenvironment by senescent cells, and despite the lack of mechanistic details thus far, it adds to the notion that, inflammation, DDR, senescence and cytokines contribute to cancer development (Coppe et al., 2008; Rodier et al., 2009).

## Pathogens and DDR

Many virulence factors induce host DNA damage and DDR activation. Viruses such as EBV, Herpes-Simplex virus 1,2 (HSV 1,2), cytomegalovirus (CMV), hepatitis C virus (HCV), HPV, are all associated with cancerous pathologies, triggering DDR activation (Georgakilas et al., 2010; Pateras et al., 2015).

Bacteria such as *Escherichia coli, Campylobacter jejuni, Streptococcus bovis*, and *H. pylori* are known to induce DNA damage in host cells (Nougayrede et al., 2006; Liyanage et al., 2010). Especially, *S. bovis* is strongly associated with colorectal cancer. It is still unknown whether bacteria or their toxins induce tumorigenesis directly by cell transformation (Cortes-Bratti et al., 2001; Scanu et al., 2015) or indirectly due to the accompanying chronic inflammation (Coussens and Werb, 2002) or whether both mechanisms operate.

## *H. pylori* and its Implication in DDR and Carcinogenesis

In 2005, the Nobel Prize in Physiology or Medicine was awarded to Barry Marshall and Robin Warren, for their discovery of the bacterium *H. pylori* and its role in gastritis and peptic ulcer disease (Marshall and Warren, 1984). The bacterium is the major cause of peptic ulcer, gastric cancer, and lymphoma of Mucosa Associated Lymphoid Tissue (MALT) (Marshall and Warren, 1984; Parsonnet et al., 1997; Parsonnet and Isaacson, 2004). *H. pylori* colonizes the gastric mucosa of almost half the world population and although provokes a robust inflammatory response of the gastric mucosa, evades eradication leading to lifelong infection.

Despite the fact that *H. pylori* is associated with persistent gastric mucosa inflammation, the great majority of the infected population (∼80%) remains asymptomatic. Three important factors are associated with the development of symptomatic *H. pylori* disease: (1) *H. pylori’s* virulence factors (2) host susceptibility and response and (3) environmental cofactors, including smoking and diet (Atherton, 2006).

### *H. pylori’s* Virulence Factors

*Helicobacter pylori’s* CagA, encoded by the *cagA* gene within the CagPAI of *H. pylori* genome, is the most important of its virulent factors. CagA(+) strains induce enhanced inflammation of the gastric mucosa and have stronger association with gastric cancer. CagA protein is injected by the bacterium through a type IV secretion system (TFSS) and hijacks the host cell molecular machinery by interfering with multiple host signaling pathways including NFκB and MAPKs (Keates et al., 2001), affecting apoptosis, cell growth and motility (Odenbreit et al., 2000; Lin et al., 2010; Murata-Kamiya, 2011). CagA(+) strains also induce higher levels of expression of the proinflammatory cytokines TNFα, IL-1β, and IL-8 that trigger oxidative stress and oxidative DNA damage in the infected mucosa, thus promoting genomic instability and tumorigenesis (Blaser et al., 1995; Peek et al., 1995; Eftang et al., 2012). Other *H. pylori* virulence factors, such as VacA and NapA, also augment the inflammatory response and oxidative stress (Kim et al., 2007).

### Host Response to *H. pylori* Infection

*Helicobacter pylori-*induced host inflammatory response rather enhances than attenuates its pathogenicity. Both innate and adaptive host responses are activated during the infection (Hardbower et al., 2014). Neutrophils and macrophages trigger ROS and NO production, create oxidative stress and tissue damage. The bacterium survives oxidative stress by the production of the enzymes Nap A, catalase and superoxide catalase; thus, leading to persistent immunocyte infiltration in the gastric mucosa and further enhancement of oxidative burst (Wilson et al., 1996; Ramarao et al., 2000).

Adaptive immune response in *H. pylori* infection is mainly mediated by Th1 and Th17 T cells that secrete INFγ and IL-17 and are thought to further induce inflammatory cells influx and perpetuate mucosal damage (D’Elios et al., 1997; Szabo et al., 1999). VacA, Cag A, gamma-glutamyltranspeptidase, and arginase produced by *H. pylori* alter T-cells responses by interfering with T cell proliferation, activation and apoptosis (Gebert et al., 2003; Gerhard et al., 2005; Wang et al., 2010; Larussa et al., 2015). Interestingly, the bacterium, through dendritic cells, can induce T-regulatory(Treg)/TGF-β activation that blunts Th1/Th17 responses, early in the course of the infection. It has been suggested that the attenuation of Treg responses during progression of the infection shifts the balance leading to more inflammation and more oxidative DNA damage (Harris et al., 2008; Kao et al., 2010; Wang et al., 2010).

Furthermore, host polymorphisms affecting cytokines and especially IL-1β, TNF-α, and IL-10 are considered as important factors of host susceptibility to peptic ulceration and gastric adenocarcinoma, linking local inflammatory responses with tumorigenesis (El-Omar et al., 2000; Machado et al., 2001; Furuta et al., 2002; Hwang et al., 2002; Rad et al., 2004). Individuals infected with more virulent strains of *H. pylori* and carriers of multiple cytokine polymorphisms are considered to be more susceptible to gastric cancer development.

### *H. pylori* Promotes Gastric Carcinogenesis

Since 1990s when epidemiologic studies provided evidence for the association between *H. pylori* and gastric cancer and the bacterium was classified as human carcinogen (Forman et al., 1991; Nomura et al., 1991; Parsonnet et al., 1991), our knowledge on the mechanisms of *H. pylori* induced carcinogenesis has been greatly improved and still evolving, due to the rigorous research in this field.

Several reports also suggest a putative role of EBV in gastric carcinogenesis, more evident in gastric stump and non antral tumors (Murphy et al., 2009). Recent data from the Cancer Genome Atlas (TCGA) project led to the classification of gastric cancer into four subtypes: EBV related, microsatellite unstable, genomically stable, and CIN tumors; each type is characterized by specific molecular defects and elevated expression of DDR pathways (Cancer Genome Atlas Research Network, 2014). Although the significance of EBV relationship to gastric carcinogenesis is largely unexplored, studies clearly show that eradication of *H. pylori* reduces the risk of gastric cancer development, especially in patients without precancerous lesions (Wong et al., 2004; Ford et al., 2014).

According to the Correa model, *H pylori* infection induced gastritis progresses to gastric cancer through the premalignant stages of gastric atrophy, IM, and dysplasia (Correa, 1992). More recent evidence suggests that *H. pylori* related IM may result from SPEM, a metaplastic epithelium developing during the infection as a result of parietal cells loss and trans-differentiation of chief cells. Persistent inflammation may advance SPEM into a more proliferative metaplasia and to adenocarcinoma development (Weis and Goldenring, 2009). Nevertheless, the majority of *H. pylori* carriers are asymptomatic and although almost 20% acquire preneoplastic changes, approximately only 2% will develop gastric cancer and lymphoma (Peek and Blaser, 2002).

Gastric carcinogenesis in *H. pylori* infection is affected by several host and bacterial virulence factors. Dysregulation of cellular homeostasis in gastric mucosa is the main result of host response to the pathogen, favoring chronic inflammation, DNA lesions, and finally tissue damage (Preston-Martin et al., 1990; Algood et al., 2007). BMDCs that migrate to gastric mucosa in the context of *H. pylori* infection may aid to the neoplastic process, although evidence is mainly from animal model studies (Houghton et al., 2004; Bessede et al., 2014).

A dysregulation of apoptosis, including its induction or inhibition may play a key-role in *H. pylori* tumorigenesis. Induction of apoptosis results to gastric atrophy, hypochlorhydria and possibly to BMDCs recruitment, promoting tumor growth. Inhibition of apoptosis attenuates defense mechanisms of host against DNA damage, accumulates genetic errors and promotes cell malignant transformation (Sougioultzis et al., 2003; Ricci et al., 2011).

It should be mentioned that both experimental and human evidence suggest that *H. pylori* also induces mitochondrial DNA (mtDNA) mutagenesis which seems to enhance oxidative stress and contribute to gastric cancer development (Machado et al., 2009).

### *H. pylori* Triggers DDR

Bartkova et al. (2005) as well as Gorgoulis et al. (2005) have demonstrated that phosphorylated 53BP1, ATM, H2AX, Chk2 and p53, are indicative of DDR activation in premalignant lesions, following DSBs formation. Xie et al. (2014) studied γH2AX expression in human gastric tissue samples, irrespective of *H. pylori* infection. The levels of γH2AX gradually increased from chronic gastritis, IM, to dysplasia and were higher in the presence of *H. pylori*. In gastric cancer γH2AX was also expressed, but in lower levels than in the aforementioned premalignant lesions (Xie et al., 2014).

Toller et al. (2011), examined the activation of DDR pathway, in transformed cells (AGS) infected with *H. pylori.* They concluded that *H. pylori* infection induces the formation of DSBs through a direct host–pathogen contact and triggers DDR pathway, as assessed by the phosphorylation of H2AX. DSBs due to *H. pylori* are continuously repaired, although prolonged infection may compromise DDR and result to unrepaired breaks (Toller et al., 2011).

Hanada et al. (2014), reported that ATM is activated in formalin fixed *H. pylori* infected human mucosa tissue samples, as well as in *H. pylori* infected AGS cultured cells. DDR activation was also confirmed by γH2AX expression in *H. pylori* infected AGS cells that co-expressed ATM. They also observed that both CagA+ and CagA- strains induce DNA damage, albeit CagA+ strains are related with a greater DNA damage and more potent DDR activity (Hanada et al., 2014).

Koeppel et al. (2015) recently reported that *H. pylori* induces a specific pattern of DNA damage in infected cells, different from other mutagenic agents, mostly affecting chromosomal ends, resulting in telomeres loss and CIN, genetic alterations that are implicated in gastric carcinogenesis. They also observed that *H. pylori* compromises DDR by inhibiting several factors involved, such as ATR, MRE11, and NBS1. In another series of experiments Hartung et al. (2015)showed that DSBs in *H. pylori* infected cells are introduced by XPF/XPG endonucleases in a type IV secretion system (T4SS)-dependent manner that requires NF-κB/RelA activation. Interestingly, DSBs induced by the bacterium promote NF-κB target gene transactivation and host cell survival (Hartung et al., 2015; Koeppel et al., 2015).

**FIGURE 1:**
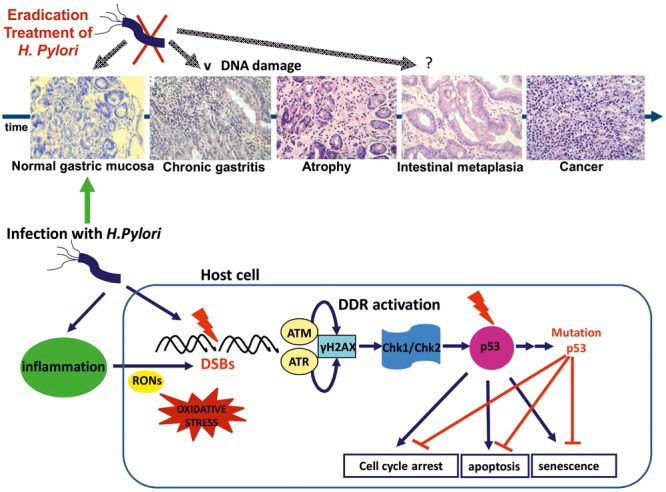
***Helicobacter pylori* infection results to DSBs possibly through a direct pathogen-host interaction.** Inflammation through oxidative/nitrosative stress seems to play a significant role. DSBs formation triggers DDR activation through ATM and ATR activation and subsequent phosphorylation of H2AX at Ser 139 (γH2AX). γH2AX formation activates the transducers Chk1 and Chk2 leading to p53 activation and promoting cell cycle arrest for DNA lesion repair or alternatively induction of apoptosis or premature senescence. DNA replication stress results to genomic instability and selective pressure for p53 mutations abrogating the tumor suppressing actions of p53 (Bartek and Lukas, 2007). *H pylori* infection induced gastritis progresses to gastric cancer through the premalignant stages of gastric atrophy, IM, and dysplasia according to the Correa model (representative histology slides, eosin-hematoxylin photos x200). *H. pylori* eradication at the early stages of gastritis and atrophy may result to attenuation of DNA damage and DDR activation. IM is considered as “a point of no return” and p53 mutations are common in the metaplastic epithelium (Ochiai et al., 1996; Xie et al., 2014).

Although limited, both *in vitro* and *in vivo* evidence suggest, that *H. pylori* infection promotes DSBs formation either directly through host–pathogen contact or indirectly due to the accompanying chronic inflammation, eventually resulting to DDR activation (**Table 1**). Persistent infection and associated inflammation may compromise the DDR pathway leading to mutations of p53, unrepaired DNA damage and even tumorigenesis. Indeed, p53 mutation is a frequent (32%) and early event in sporadic gastric cancer (Ochiai et al., 1996; Petitjean et al., 2007) (**Figure 1**).

**Table 1:** Reviewed bibliography on DDR activation and *H. pylori.*

	Reported findings	Reference
*In vitro*	*H. pylori* infected cells activate 53BP1, ATM, γH2AX/DNA damage through BabA, nor CagA, VacA or oxidative stress	Toller et al., 2011
	*H. pylori* infected cells present a specific pattern DNA damage mediated through CagPAI and oxidative stress	Koeppel et al., 2015
	*H. pylori* infection activates γH2AX/CagA+ strains present greater activity	Hanada et al., 2014
	*H. pylori* infection promotes DSBs formation through a type IV secretion system (T4SS)-dependent manner that requires NF-κB/RelA activation	Hartung et al., 2015
*In vivo*	*H. pylori* infection activates DDR through γH2AX. γH2AX expression through gastritis, intestinal metaplasia, atrophy and remains high in gastric cancer	Xie et al., 2014
*Ex vivo*	*H. pylori* infection activates ATM	Hanada et al., 2014

## Future Perspectives

There is little doubt nowadays that *H. pylori* is a risk factor for gastric cancer development. Gastric mucosa inflammation, oxidative burst and the resultant altered epithelial cell turnover are implicated. Accumulating evidence, as briefly outlined above, suggest that the bacterium causes DNA damage to the host cells and triggers DDR. Interestingly, *H. pylori* seems to compromise the integrity of DDR by affecting various proteins of the pathway and, in parallel, maintains its niche by promoting cell survival through DSBs induced NFκB gene-transactivation (Hartung et al., 2015; Koeppel et al., 2015). Hence, *H. pylori* triggers the DDR and immune response crosstalk promoting a vicious cycle of DNA damage and persistent inflammation that fuels tumorigenesis.

DNA damage repair activity seems to decrease after eradication of *H. pylori* but probably persists in areas of IM which is considered “a point of no return,” meaning that it is not reversible after *H. pylori* clearance and frequently contains p53 mutations. Studying the DDR pathway in *H. pylori* related IM with mutated vs. wild p53 will likely provide useful molecular data on the development of the metaplastic-precancerous epithelium and lead to targeted therapeutic interventions for gastric cancer prevention.

## Author Contributions

PKa, ES, IP designed the report. PKa, ES, IP, AV, and IK reviewed the bibliography. PKa and ES collected the data and wrote the paper. PKa, ES, IP, and VG performed the immunohistochemical analysis. PKo performed the pathological analysis. SS, PV, and VG supervised the manuscript.

## Conflict of Interest Statement

The authors declare that the research was conducted in the absence of any commercial or financial relationships that could be construed as a potential conflict of interest.

## Abbreviations

BMDCs: bone marrow derived cells
CIN: chromosomal instability
DDR: DNA damage repair
DSBs: double strand breaks
EBV: Epstein–Barr virus
*H. pylori*: *Helicobacter pylori;*
IM: intestinal metaplasia
MDSCs: myeloid derived suppressor cells
NBS: Nijmegen breakage syndrome
RONS: reactive oxygen and nitrogen species
SASP: senescence-associated secretory phenotype
SSBs: single strand breaks
SPEM: spasmolytic polypeptide expressing metaplasia
Treg: T-regulatory cells
γH2AX: phosphorylated H2AX
